# Identification and characterisation of two high-affinity glucose transporters from the spoilage yeast *Brettanomyces bruxellensis*

**DOI:** 10.1093/femsle/fnz222

**Published:** 2019-10-30

**Authors:** Ievgeniia A Tiukova, Iben Møller-Hansen, Zeinu M Belew, Behrooz Darbani, Eckhard Boles, Hussam H Nour-Eldin, Tomas Linder, Jens Nielsen, Irina Borodina

**Affiliations:** 1 Division of Systems and Synthetic Biology, Department of Biology and Biological Engineering, Chalmers University of Technology, Kemigården 4, 412 96 Gothenburg, Sweden; 2 The Novo Nordisk Foundation for Biosustainability, Technical University of Denmark, Building 220, 2800 Kongens Lyngby, Denmark; 3 Department of Plant and Environmental Sciences, DynaMo Center, Copenhagen Plant Science Center, University of Copenhagen, Thorvaldsensvej 40, 1871 Frederiksberg, Denmark; 4 Institute of Molecular Biosciences, Faculty of Biological Sciences, Goethe University Frankfurt, Max-von-Laue Straße 9, 60438, Frankfurt am Main, Germany; 5 Department of Molecular Sciences, Swedish University of Agricultural Sciences, Almas allé 5, 750 07 Uppsala, Sweden

**Keywords:** bioethanol, glucose transport, metabolism, high-affinity, *Brettanomyces bruxellensis*, *Xenopus laevis*, yeast

## Abstract

The yeast *Brettanomyces bruxellensis* (syn. *Dekkera bruxellensis*) is an emerging and undesirable contaminant in industrial low-sugar ethanol fermentations that employ the yeast *Saccharomyces cerevisiae*. High-affinity glucose import in *B. bruxellensis* has been proposed to be the mechanism by which this yeast can outcompete *S. cerevisiae*. The present study describes the characterization of two *B. bruxellensis* genes (*BHT1* and *BHT3*) believed to encode putative high-affinity glucose transporters. *In vitro*-generated transcripts of both genes as well as the *S. cerevisiae HXT7* high-affinity glucose transporter were injected into *Xenopus laevis* oocytes and subsequent glucose uptake rates were assayed using ^14^C-labelled glucose. At 0.1 mM glucose, Bht1p was shown to transport glucose five times faster than Hxt7p. pH affected the rate of glucose transport by Bht1p and Bht3p, indicating an active glucose transport mechanism that involves proton symport. These results suggest a possible role for *BHT1* and *BHT3* in the competitive ability of *B. bruxellensis*.

## Teaser

Identification and characterization of two high-affinity glucose transporters from the spoilage yeast *Brettanomyces bruxellensis*.

## INTRODUCTION

Glucose is a key source of energy and carbon for many heterotrophic organisms and consequently it is also an important global regulator of cell metabolism. The import of glucose into eukaryotic cells is perhaps best understood in the common baker's yeast *Saccharomyces cerevisiae* not only due to its common use as a genetic model organism but also because of the central importance of yeast glucose import in the production of alcoholic beverages as well as bioethanol (Boles 2003). The *S. cerevisiae* genome contains around 20 transporters capable of glucose import, which are encoded by the genes *HXT1*-*HXT17, GAL2, AGT1, MPH3* and *YDL247W* (Wieczorke et al. 1999). Glucose import in batch-grown *S. cerevisiae* is mainly mediated by the transporters Hxt1p, Hxt2p, Hxt3p, Hxt4p, Hxt6p and Hxt7p (Reifenberger and Freidel 1995). However, it has been shown that individual overexpression of the remaining 11 glucose transporters of the *HXT* family (with the exception of the pseudogene *HXT12*) can sustain growth in a *HXT1*-*HXT7* hexose transporter-null (*hxt0*) mutant strain when cultivated on glucose or fructose (Wieczorke et al. 1999).

The expansion of glucose transporter genes in the *S. cerevisiae* genome through gene duplication is thought to be an adaptation that enables this species to grow efficiently over a wide range of glucose concentrations. The *S. cerevisiae* genes encoding low affinity glucose transporters (*HXT1, HXT3, HXT4, HXT5*; K_m_ 9–100 mM) are expressed at higher-glucose concentrations, while expression of genes encoding high-affinity glucose transporters (*HXT2, HXT6, HXT7*; K_m_ 1.5 mM) is upregulated when glucose levels drops below 5 mM (Reifenberger, Boles and Ciriacy 1997).

*S. cerevisiae* is widely used not only in food and beverage production but it is also a favored cell factory for various industrial biotechnology applications. Despite its arsenal of hexose transporters, *S. cerevisiae* remains vulnerable to microbial contaminants during continuous fermentations at near zero-glucose levels. The spoilage yeast *Brettanomyces bruxellensis* (syn. *Dekkera bruxellensis*) is a persistent concern for the winemaking industry, where this yeast tends to dominate during secondary fermentations, when only minute amounts of sugar are available (Steensels et al. 2015; Smith and Divol 2016). More recently, *B. bruxellensis* has been found to outcompete *S. cerevisiae* inocula in industrial ethanol plants employing glucose-limited continuous fermentation (de Souza Liberal et al. 2007; Passoth, Blomqvist and Schnürer 2007). It has been suggested that efficient high-affinity glucose import is what mediates the competitive advantage of *B. bruxellensis* over *S. cerevisiae* under condition of glucose limitation (Tiukova et al. 2013).

Respiratory (Crabtree negative) yeasts, such as *Cyberlindnera jadinii, Kluyveromyces marxianus, Ogataea nonfermentans* and *Scheffersomyces stipitis* have been shown to transport glucose with 10–50 times higher affinity (2–200 μM) than that of *S. cerevisiae* (van Urk et al. 1989). While glucose import in *S. cerevisiae* is passive facilitated diffusion (Maier et al. 2002; Boles 2003), high-affinity glucose import in some Crabtree negative yeasts may proceed via an active proton (H^+^) symport mechanism (van Urk et al. 1989). High-affinity sugar import through proton symport enables sugar import against a concentration gradient. *Scheffersomyces stipitis* has been shown to utilise proton symport for both glucose and xylose uptake (Kilian and van Uden 1988). A fructose symporter has been described in the lager yeast *Saccharomyces pastorianus* (Gonçalves, Rodrigues de Sousa and Spencer-Martins 2000). Intracellular pH is maintained through compensatory proton export using the plasma membrane H^+^-ATPase (Weusthuis et al. 1993). Notably, although *B. bruxellensis* is a fermentative (Crabtree positive) yeast similarly to *S. cerevisiae*, it also utilises active glucose import (Silva, Cardoso and Gerós 2004). Previous studies have shown that the affinity and rate of the high-affinity component of glucose transport in *B. bruxellensis* cells corresponded to K_m_ = 0.03 mM and V_max_ = 0.32 nmol glucose s^–1^ mg dry cell weight^–1^ when cultivated in 1 g l^–1^ glucose (Silva, Cardoso and Gerós 2004). The kinetics of the high-affinity component of glucose transport in *S. cerevisiae* grown in 2 g l^–1^ glucose corresponded to K_m_ = 1.6 mM and V_max_ = 162 nmol min^–1^ mg of protein^–1^ (Walsh et al. 1994).

A previous transcriptome study of *B. bruxellensis* CBS  11270 cultivated in sugar-limited conditions had identified two highly expressed genes, which were hypothesised to encode high-affinity glucose transporters based on homology analysis (Tiukova et al. 2013). The present study sought to characterize these previously identified candidate transporter genes from *B. bruxellensis* with the help of the *Xenopus laevis* oocyte expression system (Tammaro, Shimomura and Proks 2008). Oocytes from the *X. laevis* represent one of the most widely used systems for heterologous expression and characterisation of membrane transporters. Notably, *X. laevis* oocytes provide a robust and versatile alternative expression system to yeast cells for the characterisation of transporter proteins. For example, difficulties have previously been reported for the heterologous expression of rat glucose transporters GLUT1 and GLUT4 in a *S. cerevisiae hxt0* strain (Kasahara and Kasahara 1997). *X. laevis* oocytes have previously been used to successfully characterise hexose transporters from a wide spectrum of organisms including human (Gould et al. 1991; Rogers et al. 2003), the apicomplexan parasite *Babesia bovis* (Derbyshire et al. 2008), and plant *Arabidopsis thaliana* (Nour-Eldin, Nørholm and Halkier 2006). Therefore, the *X. laevis* oocyte expression system was selected in the present study to characterise putative *B. bruxellensis* hexose transporters.

## MATERIALS AND METHODS

### Sequence retrieval and phylogenetic analysis

In the present study, putative *B. bruxellensis* genes encoding glucose transporters were identified through TBLASTN searches against the GenBank wgs database constrained to *B. bruxellensis* (taxid: 5007) using query sequences indicated in the text. Protein sequences were translated from genomic DNA using the NCBI ORFfinder server (https://www.ncbi.nlm.nih.gov/orffinder/). Predicted protein sequences were aligned in MAFFT (Katoh et al. 2005; http://mafft.cbrc.jp/alignment/server/index.html) using the G-INS-i alignment strategy. Aligned sequence positions suitable for phylogenetic analysis were selected using GBlocks (Castresana 2000; http://molevol.ibmb.csic.es/Gblocks_server/) with the settings for smaller final blocks and less strict flanking positions enabled. The trimmed amino acid data set was then used to construct a maximum likelihood tree using PhyML v. 3 (Guindon et al. 2010; http://www.atgc-montpellier.fr/phyml/) with 1000 bootstrap replicates. The evolutionary model for the phylogenetic analysis was estimated using SMS (Lefort, Longueville and Gascuel 2017). The resulting tree was visualised using FigTree v. 1.4.2 (http://tree.bio.ed.ac.uk/software/figtree/).

### Cloning and *in vitro* transcription of yeast glucose transporter genes

The *BHT1, BHT2* and *BHT3* genes were PCR amplified from *B. bruxellensis* CBS 11270 genomic DNA using the primers listed in Table 1. The *HXT7* gene was PCR amplified from *S. cerevisiae* prototrophic CEN.PK 113–7D (*MATa URA3 HIS3, LEU2 TRP1 MAL2-8c SUC2*) genomic DNA using the primers listed in Table 1. The amplified gene sequences were digested with USER enzyme (New England Biolabs Inc.) and inserted into PacI/Nb.BsmI-digested pNB1u plasmid (Darbani et al. 2016) downstream of the T7 promoter.

**Table 1.** Primers used in this study

Primer name	Sequence (5′ → 3′)
*BHT1* EC fwd	AGTGCAGGUAAAACAATGTCATCTTCTTCTGAGAT
*BHT1* EC rev	CGTGCGAUTCAAGCGTAGTCTGGAACGTCGTATGGGTATCCACCTCCACCTCCACCAGCCTTCTGGGCGACCTCTCCC
*BHT2* EC fwd	AGTGCAGGUAAAACAATGTCTTCCTCGGAAATTAG
*BHT2* EC rev	CGTGCGAUTCAAGCGTAGTCTGGAACGTCGTATGGGTATCCACCTCCACCTCCACCGAAAACTTTGAGGAAGGCAGTA
*BHT3* EC fwd	AGTGCAGGUAAAACAATGTCATCTTCAGAGATTAG
*BHT3* EC rev	CGTGCGAUTCA AGCGTAGTCTGGAACGTCGTATGGGTATCCACCTCCACCTCCACCAGCTTTCTGAGCTGTCTCTTCT
*HXT7* EC fwd	AGTGCAGGUAAAACAATGTCACAAGACGCTGCTA
*HXT7* EC rev	CGTGCGAUTCAAGCGTAGTCTGGAACGTCGTATGGGTATCCACCTCCACCTCCACCTTTGGTGCTGAACATTCTCTTG
*CASSETTE* fwd	GTGCTGCAAGGCGATTAAGTTGGGTAACGC
*CASSETTE* rev	CCTCGAGGCGGCCGCCTGCAG

Linear templates for *in vitro* transcription were generated by PCR amplification of the yeast gene expression cassettes within the pNB1u plasmids using primers *CASSETTE* fwd and *CASSETTE* rev. Capped complementary RNA (cRNA) of *BHT1, BHT2, BHT3, HXT7* and GFP was synthesized *in vitro* using the mMESSAGE mMACHINE^®^ T7 Transcription Kit (ThermoFisher). RNA quality and integrity was confirmed by BioAnalyzer using RNA 6000 Nano kit according to the manufacturer's instructions (Agilent Technologies, Germany) (see Fig. S1A, Supporting Information). Some instances of premature termination of *BHT2, BHT3* and *HXT7* cRNA synthesis were observed and could be caused by stretches of sequence that resemble the T7 phage polymerase termination signals or other sequence elements.

### Oocyte glucose import assays

*X. laevis* oocytes were purchased from EcoCyte Bioscience (Castrop-Rauxel, Germany). For oocyte expression, 50 nl of full-length *in vitro* synthesized cRNA of the glucose transporter genes (400 ng μl^–1^) were co-injected with cRNA of GFP (100 ng μl^–1^) into oocytes using a fully-automated roboinject system (Multi Channel Systems MCS GmbH) as described previously (Hogg et al. 2008). In this study, we used two Kulori buffers with pH 5 (90 mM NaCl, 1 mM KCl, 1 mM CaCl_2_, 1 mM MgCl_2_ and 10 mM MES) and pH 7.4 (90 mM NaCl, 1 mM KCl, 1 mM CaCl_2_, 1 mM MgCl_2_ and 10 mM HEPES). Oocytes were incubated for 3 days in Kulori buffer pH 7.4, before performing glucose transport assays. Sufficient expression levels in the injected oocytes were determined by detecting the fluorescence from GFP using a microplate reader (Synergy MX, BioTek).

Just prior to the glucose import assay, approximately seven oocytes per individual assay were washed with Kulori buffer and then pre-incubated for 5 min in 500 μl Kulori buffer at a pH similar to that used for the uptake assay (either pH 7.4 or pH 5). Oocytes expressing the same transporter was pooled and transferred to a single well in a 96-well plate with round bottom. The assay was initiated by adding 150 μl Kulori buffer containing 3 μCi ml^–1^^14^C-labelled glucose (275.0 mCi/mmol, PerkinElmer). The final concentration of glucose was adjusted to 10 μM, 0.1 mM or 1 mM by adding unlabelled glucose. After 1 hour of incubation, the assay was stopped by the addition of Kulori buffer. All intact oocytes were washed three times in Kulori buffer. Individual oocytes were then transferred to scintillation vials and lysed in 100 μl 10% (w/v) SDS by vortexing. About 2.5 ml of EcoScint^TM^ scintillation fluid (National Diagnostics) was subsequently added to each vial followed by vortexing. Radioactivity was quantified using liquid scintillation counting. Statistical significant differences between the means of glucose uptake rates were determined using a student's *t*-test.

### Western blot analysis

Western blot analysis was performed as described earlier (Jørgensen, Nour-Eldin and Halkier 2016). Coding sequences carrying C-terminal HA-tags were identified using antibodies against HA-tag (Abcam). Equal loading was confirmed using antibodies against actin (Abcam).

### Expression of the glucose transporter genes *BHT1, BHT2, BHT3* and *HXT7* in *S. cerevisiae* EBY.VW4000

The coding sequences of *BHT1, BHT2, BHT3* and *HXT7* as well as the *S. cerevisiae PGK1* promoter sequence were PCR amplified using the corresponding primers listed in Table 1. Amplification products were digested with USER enzyme (New England Biolabs Inc.) (Nour-Eldin, Nørholm and Halkier 2006) and inserted into SfaAI/Nb.BsmI-digested p0054 plasmid (pESC-URA 2 micron episomal plasmid containing *URA3* marker for selection in *S. cerevisiae*) (Jensen *et al.*^2014^). The *S. cerevisiae* EBY.VW4000 strain, which lacks the glucose transporter-encoding genes *(Δhxt1-Δhxt17 Δgal2 Δstl1 Δagt1 Δmph2 Δmph3*) (Wieczorke et al. 1999), was transformed with individual plasmids containing the transporter-encoding genes using the lithium acetate method (Gietz and Woods 2002). Positive transformants were selected for on solid SC medium lacking uracil (Sigma-Aldrich) with 20 g l^–1^ maltose as the sole carbon source.

### Profiling of growth of *S. cerevisiae* EBY.VW4000 expressing either *BHT1, BHT2, BHT3* or *HXT7*

Each yeast strain was pre-cultured in baffled 500-ml Erlenmeyer flasks with 150 ml medium. The medium was SC medium without uracil with 20 g l^–1^ maltose as the sole carbon source. For the cultivation of the control strain, we supplemented the uracil. The flasks were incubated overnight in a rotary shaker-incubator set to 30°C with 200 rpm agitation. The cultures were used as inoculum. The glucose growth assay was performed in polypropylene square 96-deepwell microplates with laser-welded bottom (CR1496d, System Duetz). Individual strains were inoculated at an initial OD600 of 0.1 in 0.5 ml of SC medium lacking uracil with the exception of the EBY.VW4000 strain control, which was supplemented with 20 mg l^–1^ uracil. Glucose was included as the sole carbon source at a concentration of either 20 or 2 g l^–1^. Incubation and automated cell density measurements were performed using an EnzyScreen 960 growth profiler set to 30°C with 220 rpm agitation.

## RESULTS AND DISCUSSION

### Identification of genes encoding putative glucose transporters in the *B. bruxellensis* genome

No glucose transporters have been characterized in *B. bruxellensis* to date. A previous transcriptome study of *B. bruxellensis* CBS 11270 had identified two putative high-affinity glucose transporters that displayed high-transcript levels under low glucose conditions, where this strain had been shown to outcompete *S. cerevisiae* (Tiukova et al. 2013). At the time, no genome sequence was yet available for the CBS 11270 strain of *B. bruxellensis* and so the closest hits of the transcript reads were two annotated proteins (GenBank protein accessions EIF46974 and EIF46975) in the genome of *B. bruxellensis* strain AWRI1499.

**Figure 1.**
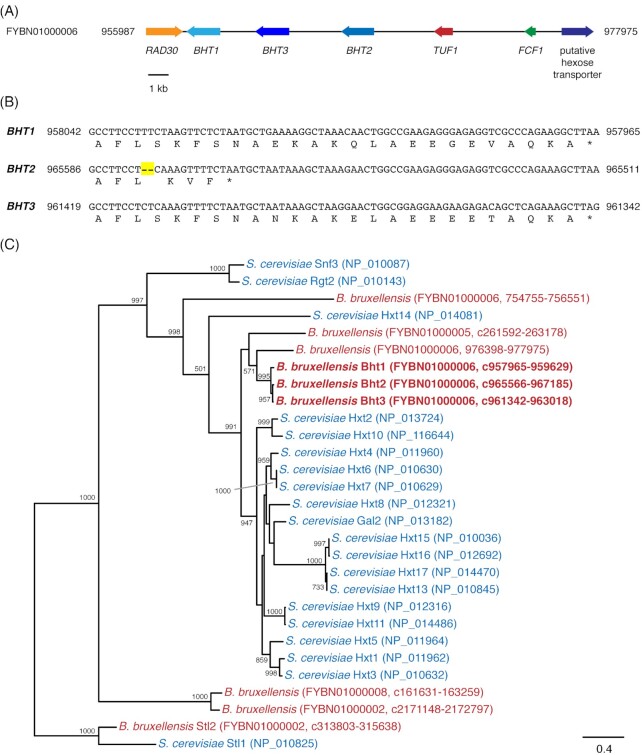
Identification and characterization of hypothetical genes encoding putative glucose transporters in the genome of *B. bruxellensis*. **(A)**, Genomic context of the *BHT1*-BHT3 gene cluster in the genome of *B. bruxellensis* UMY321. Gene names of adjacent genes were assigned based on sequence homology to previously described genes in *S. cerevisiae*. **(B)**, Alignment of the 3’ termini of the *B. bruxellensis* hypothetical genes *BHT1, BHT2* and *BHT3*. The proposed 2-bp deletion in the *BHT2* sequence is indicated in yellow. Genomic coordinates of the source contig (GenBank accession FYBN01000006) are indicated. **(C)**, Maximum likelihood tree of predicted glucose transporters in *S. cerevisiae* strain S288C (blue) and *B. bruxellensis* strain UMY321 (red) using 423 aligned amino acid positions. Node stability is indicated by the bootstrap value of 1000 replicate analyses. Bootstrap values below 500 are not shown. The *S. cerevisiae* glycerol transporter Stl1p and its *B. bruxellensis* ortholog (Zemancíková et al. 2018) were used as outgroups. GenBank protein accession numbers for *S. cerevisiae* transporters are displayed. For *B. bruxellensis* hypothetical genes, the genomic contig GenBank accession number and genomic coordinates used for conceptual translation are indicated (‘c’ signifies that the hypothetical gene is encoded on the reverse strand). The three *B. bruxellensis* putative glucose transporters *BHT1*-*BHT3* are highlighted in bold font.

Following the subsequent sequencing of *B. bruxellensis* strain CBS 11270 (Tiukova et al. 2019), TBLASTN searches against the genome using the predicted protein sequences from strain AWRI1499 identified a 20-kb segment within a genomic contig (GenBank accession UFQA01000418) of the CBS 11270 genome that appeared to contain the putative glucose transporters. However, due to stretches of ambiguous sequence in the CBS 11270 contig, it was not possible to resolve complete open reading frames. A TBLASTN search of the genome of the closely related *B. bruxellensis* strain UMY321 (Fournier et al. 2017) managed to identify the corresponding intact genomic region (GenBank accession FYBN01000006). Subsequent analysis found three hypothetical single-exon genes arranged in tandem whose predicted protein sequences were more than 90% identical to the query sequences from strain AWRI1499. The three genes were named *BHT1* (for putative *Brettanomyces h*exose *t*ransporter *1*; GenBank accession FYBN01000006, reverse complement of residues 959 629–957 965), *BHT2* (GenBank accession FYBN01000006, reverse complement of residues 967 185–965 566) and *BHT3* (GenBank accession FYBN01000006, reverse complement of residues 963 018–961 342), respectively (Fig. 1A). A fourth putative hexose transporter was detected approximately 9 kb distal to the *BHT2* gene in the *B. bruxellensis* UMY321 genome but the predicted protein sequence of this hypothetical gene appeared to be more divergent (62% amino acid identity) to the AWRI1499 query sequences.

The genomes of four other *B. bruxellensis* strains were queried with the predicted protein sequences of *BHT1*-*BHT3* in order to investigate the intra-species conservation of this cluster of putative glucose transporters. However, the majority of currently available *B. bruxellensis* genomes did not contain contigs of sufficient length or sequence quality to be able to draw any firm conclusions. TBLASTN searches of available genomes of the related species *Brettanomyces anomalus* and *Brettanomyces custerianus* using the predicted Bhtp protein sequences revealed two hypothetical *BHT* genes in the *B. anomalus* genome.

The predicted protein sequences of all three hypothetical *B. bruxellensis BHT* genes displayed high (93%–96%) amino acid similarity to each other. However, multiple sequence alignment of the three predicted protein sequences suggested that the C-terminus of the hypothetical *BHT2* gene product appeared to be slightly truncated compared to those of *BHT1* and *BHT3*. By juxtaposing both genomic DNA and predicted protein sequences we identified a likely 2-bp deletion in 3’ end of the *BHT2* gene (Fig. 1B). This deletion was expected to result in a frame-shift and premature translational termination corresponding to a 19-amino acid truncation of the Bht2p C-terminus compared to the predicted protein sequences of Bht1p and Bht3p.

As the UMY321 strain appeared to be one of the higher quality genomes of *B. bruxellensis* currently available, the analysis was extended to the entire UMY321 genome in order to identify additional glucose transporters in this strain. At least five additional putative genes were identified in the genome of *B. bruxellensis* UMY321 that displayed high to moderate sequence similarity to the query sequences from *B. bruxellensis* strain AWRI1499. Phylogenetic analysis was carried out to reconstruct the evolutionary history of putative glucose transporters in *B. bruxellensis* UMY321 and contrast it with the *HXT* genes in *S. cerevisiae*. The resulting phylogenetic tree (Fig. 1C) showed that the predicted protein sequences of *BHT1*-*BHT3* formed a well-supported monophyletic clade adjacent to a well-supported *S. cerevisiae*-only clade containing Gal2p and all Hxt proteins except Hxt14p. Three additional hypothetical *B. bruxellensis* genes were located in close proximity of the Bht1p-Bht3p clade and the *S. cerevisiae* Gal2p and Hxt sequences, which suggests a potential role in hexose transport for these three hypothetical *B. bruxellensis* genes as well.

### Cloning of putative high-affinity glucose transporters from *B. bruxellensis* CBS 11270

Using the *B. bruxellensis* UMY321 genome sequence as guide, the hypothetical genes *BHT1*-*BHT3* were amplified by PCR from *B. bruxellensis* CBS 11270 genomic DNA, inserted into cloning plasmids and sequenced. Orthologs of *BHT1* and *BHT3* displayed 97% and 98% sequence identity, respectively, between *B. bruxellensis* strains UMY321 and CBS 11270. The sequencing of the CBS 11270 ortholog of *BHT2* proved unexpectedly problematic and only the first 1072 bases could be confidently assigned. No obvious reason for this result could be found. *In vitro* transcription of C-terminally HA-tagged variants of all three *BHT* genes and the *S. cerevisiae HXT7* gene resulted in detectable products following cRNA injection into *X. laevis* oocytes with the exception of *BHT2* (see Fig. S1B, Supporting Information). The *B. bruxellensis* CBS 11270 *BHT2* gene was therefore not characterized further in the present study. It is possible that the C-terminal truncation in the predicted protein sequence of the *BHT2* product could lead to protein aggregation and targeted proteolysis. Since the 3’ terminus of the CBS 11270 *BHT2* gene could not be resolved in the scope of this study, it remains unclear whether the same 2-bp deletion occurs in this strain as well. However, efforts are currently underway to resolve the sequence of the final 3’ portion of the *B. bruxellensis* CBS 11270 hypothetical *BHT2* gene.

### Concentration-dependent effects of glucose uptake rate in *X. laevis* oocytes expressing yeast transporters

The glucose affinities of Bht1p and Bht3p were investigated after injection of *X. laevis* oocytes with *in vitro* synthesized cRNA of the corresponding gene. The injected oocytes were incubated in Kulori buffer (pH 7.4) for 3 days and then assayed in medium containing either 0.01, 0.1 or 1 mM glucose. The *S. cerevisiae HXT7* gene, which encodes a high-affinity glucose transporter, was *in vitro* transcribed and injected in parallel for comparison. Injection of GFP cRNA was used as a control for endogenous glucose uptake by the oocytes in the absence of yeast transporters. The accumulation of ^14^C-labelled glucose in oocytes during 1 hour of incubation in Kulori buffer (pH 5) with the indicated glucose concentration was then quantified using liquid scintillation counting.

**Figure 2.**
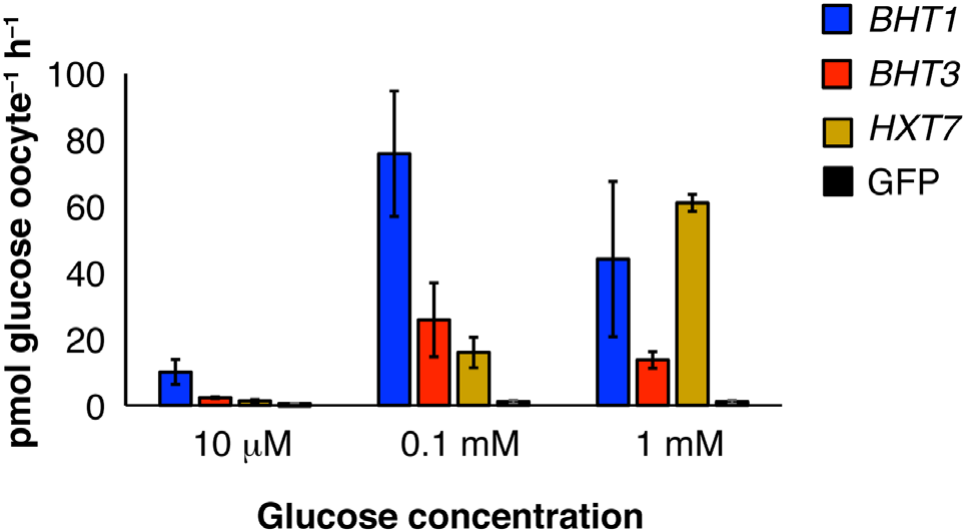
Concentration-dependent effects on glucose uptake in *X. laevis* oocytes injected with cRNA of yeast genes encoding glucose transporters. Assays were performed at pH 5 with the indicated glucose concentration. Injection of cRNA GFP was used as negative controls to account for endogenous glucose uptake. The assays were performed at least in triplicate. Error bars indicate one standard deviation.

The glucose transport rate was lowest at 0.01 mM for all tested transporters (Fig. 2). Bht1p and Bht3p displayed higher transport activity at low glucose concentrations (0.01 and 0.1 mM) compared to Hxt7p. At 0.01 and 0.1 mM glucose, Bht1p displayed the highest transport rate of the tested transporters and transported five times more glucose as compared to Hxt7p at 0.1 mM glucose. Conversely, Hxt7p displayed the highest observed glucose transport rate at the highest tested glucose concentration (1 mM). Hxt7p transport activity increased in relation to glucose concentration and although its saturation limit was not observed in this experiment, the data is in agreement with the previously reported K_m_ value of 1.5 mM for this transporter (Reifenberger, Boles and Ciriacy 1997).

^14^C-labelled glucose levels in oocytes expressing either Bht1p or Bht3p increased by a factor of 7 and 11, respectively, as external glucose concentrations increased from 0.01 to 0.1 mM. However, a further increase of external glucose concentration to 1 mM did not result in increased transport by either Bht1p or Bht3p, which would indicate that both transporters reached saturation at external glucose concentrations below 1 mM. Bht1p and Bht3p therefore appear to have higher-glucose affinity than Hxt7p with Bht1p having the highest activity.

### pH-dependent effects on glucose uptake rate in *X. laevis* oocytes expressing yeast transporters

**Figure 3.**
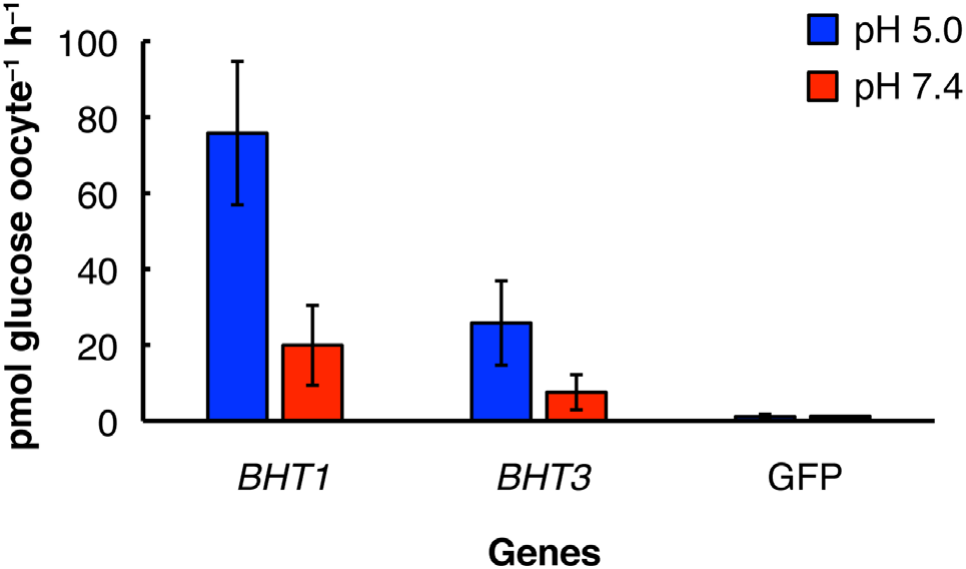
pH-dependent effects on glucose uptake in *X. laevis* oocytes injected with cRNA of *B. bruxellensis*genes encoding glucose transporters. Oocytes were incubated 1 hour in 0.1 mM glucose buffer at the indicated pH. Injection of cRNA GFP was used as negative controls to account for endogenous glucose uptake. The assays were performed at least in triplicate. Error bars indicate one standard deviation.

The effects of pH on the rate of glucose transport by Bht1p and Bht3p were tested by incubating the oocytes in a medium containing 0.1 mM glucose at either pH 5 or 7.4. The glucose transport rates of Bht1p and Bht3p were respectively 3.8 and 3.4-fold higher at pH 5 than at pH 7.4 (Fig. 3). The observed pH-dependent increase in glucose transport rate suggests an active glucose transport mechanism that involves proton symport. This raises the possibility that Bht1p and Bht3p are involved in active high-affinity proton symport glucose transport, which has previously been observed in *B. bruxellensis* (Silva, Cardoso and Gerós 2004).

### Expression of glucose transporter genes *BHT1, BHT2, BHT3* and *HXT7* in *S. cerevisiae* EBY.VW4000

The EBY.VW4000 parental strain as well as the EBY.VW4000 strain expressing *BHT2* did not grow in medium supplied with glucose as a sole carbon source. The observed failure of the *BHT2*-expressing EBY.VW4000 strain to grow on glucose was consistent with results of oocyte experiment, which suggested that the apparent C-terminal truncation of the *BHT2* gene product results in a non-functional protein. The EBY.VW4000 strain expressing *HXT7* displayed the fastest growth of all the tested transporter proteins in the present study. Expression of the *BHT1* gene in strain EBY.VW4000 resulted in a faster growth than the *BHT3* gene, which was consistent with the observations of glucose uptake in the oocytes injected with the corresponding cRNAs.

### Concluding remarks

Contamination of industrial fermentations by undesirable yeasts and bacteria is a significant source of economic losses in microbial biotechnology (Beckner, Ivey and Phister 2011; Tiukova, Eberhard and Passoth 2014). In the case of industrial bioethanol fermentations, the sugar substrate has been estimated to contribute upwards of 70% of production costs (Pfromm et al. 2010). Understanding the mechanisms of sugar uptake in the common spoilage yeast *B. bruxellensis* and other microbial contaminants is therefore of great interest for the optimization of both production organisms and cultivation parameters of low-sugar industrial fermentations in order to minimize the risk of outcompetition of the initial inoculum. In addition, the ability to efficiently acquire external nutrients across a broad range of concentrations is an attractive trait for the engineering of improved production organisms (Hara et al. 2017).

The present study focused on two *B. bruxellensis* genes—*BHT1* and *BHT3*, which were predicted to encode high-affinity glucose transporters and may therefore play a role in the ability of this yeast to outcompete *S. cerevisiae* at low sugar concentrations. The results presented here indicate that both genes do in fact encode high-affinity glucose transporters that utilise a proton symport mechanism. This study also identified a number of additional genes is *B. bruxellensis* that are predicted to encode hexose transporters (Fig. 1C), which will be the subject of future studies.

The substitution of facilitated diffusion transport by proton symporters can be utilized as metabolic engineering strategy to reduce biomass production and direct carbon utilization towards increased product formation (de Kok et al. 2012). For example, it has been suggested that replacing of the native Hxt transporters in *S. cerevisiae* with a hexose/proton symport uptake mechanism should decrease the biomass yield of anaerobic yeast cultures on hexose sugars by 50%, with a concomitant increase in the ethanol yield of 14% (Basso et al. 2011). This is in agreement with observations of both lower biomass production and higher ethanol yields in *S. cerevisiae* cultivated on maltose (Weusthuis et al. 1993) or sucrose (Basso et al. 2011) as carbon sources, which both employ proton symport transporters.

**Figure 4.**
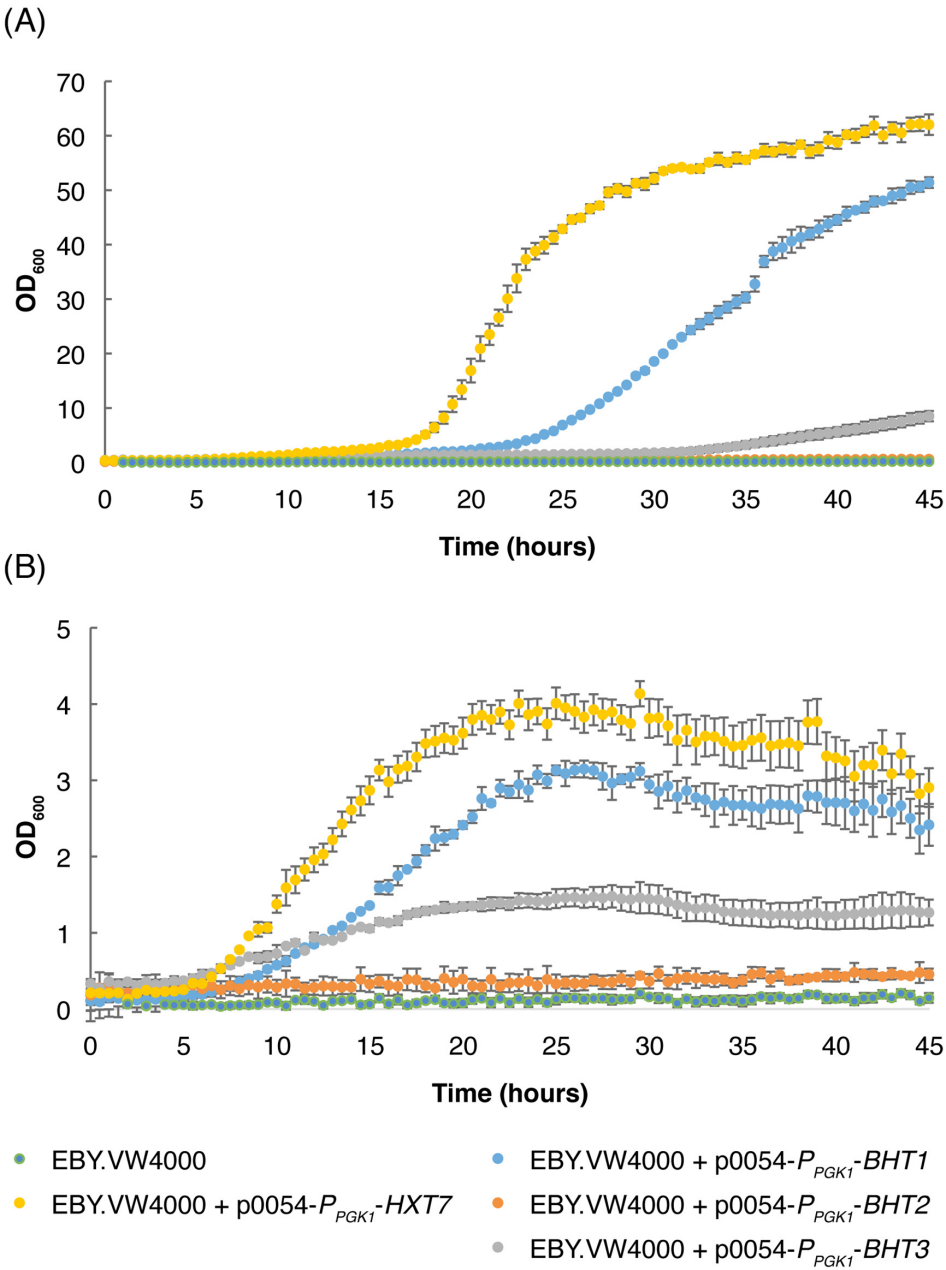
Growth profiles of a *S. cerevisiae hxt0* strain expressing individual hexose transporter genes. Cultivations were carried out in SC medium containing either 20 g l^–1^**(A)** or 2 g l^–1^**(B)** glucose. Growth assays were performed in triplicate with error bars indicating one standard deviation.

Overall, the results from the glucose uptake assays in the oocyte system (Fig. 2) agreed well with the yeast growth profile assays (Fig. 4). The pH-dependent effects on oocyte glucose uptake injected with *BHT3* cRNA suggests that this gene encodes a high-affinity glucose proton symporter.

In closing, the use of proton-dependent glucose import by *B. bruxellensis* may appear expensive from an energetics perspective when compared to the other members of the *SLC2* family identified as facilitators (Darbani, Kell and Borodina 2018; Marques et al. 2018; Darbani et al. 2019). However, this property gives this yeast a competitive edge in nutrient poor environments. Previous work suggests that *B. bruxellensis* has evolved an energy-efficient metabolism that may cover the energetic expenses of active high-affinity glucose transport. This includes low production of exported byproduct glycerol in *B. bruxellensis*. Unlike *S. cerevisiae, B. bruxellensis* has also retained respiratory complex I despite being a Crabtree positive yeast (Woolfit et al. 2007). The expression of complex I under oxygen-limited conditions would suggest different number of protons translocated during respiration and thus potentially higher ATP recovery by the respiratory chain in *B. bruxellensis* compared to *S. cerevisiae* (Tiukova et al. 2013).

## Supplementary Material

fnz222_Supplemental_Files
