# Supplementary material for: Identification and characterisation of two high-affinity glucose transporters from the spoilage yeast Brettanomyces bruxellensis
Source: FEMS Microbiol Lett. 2019 Oct 30;366(17):fnz222. doi: 10.1093/femsle/fnz222 (PMC6847091; doi:10.1093/femsle/fnz222)

Figure 1A. Electrophoregram of *in vitro* synthesized cRNA of *BHT1, BHT2, BHT3* and *HXT7*.
Premature termination of *BHT2, BHT3* and *HXT7* transcription.

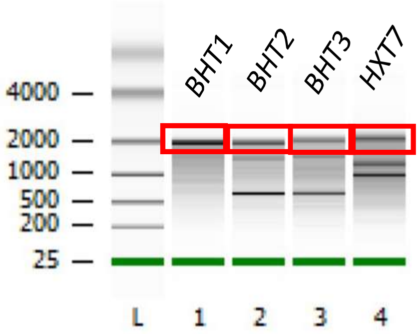

Figure 1B. Western blot analysis of proteins extracted from oocytes injected with either *BHT1* or *BHT2* or *BHT3* or *HXT7* or *GFP* cRNA and non-injected oocytes. Expression of full-size transporters was detected in oocytes injected with either *BHT1* or *BHT3* or *HXT7* cRNA. *BHT2* cRNA was not translated.

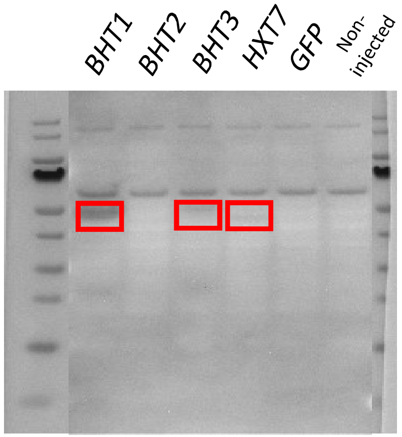

Supplement: fnz222_Supplemental_Files [file fnz222_supplemental_files.zip › Supplementary_Figure1.pdf]
